# Combination Systemic Therapies in Advanced Well-Differentiated Gastroenteropancreatic Neuroendocrine Tumors (GEP-NETs): A Comprehensive Review of Clinical Trials and Prospective Studies

**DOI:** 10.3390/biology12081069

**Published:** 2023-07-30

**Authors:** Leonidas N. Diamantopoulos, Markos Kalligeros, Thorvardur R. Halfdanarson, Nikolaos Diamantis, Christos Toumpanakis

**Affiliations:** 1 Department of Medicine, University of Pittsburgh Medical Center, 200 Lothrop Street, Pittsburgh, PA 15213, USA; diamantopoulosln@gmail.com; 2 Department of Medicine, Warren Alpert Medical School of Brown University, Providence, RI 02903, USA; markos_kalligeros@brown.edu; 3 Department of Medicine, Division of Hematology/Oncology, Mayo Clinic, Rochester, MN 55905, USA; halfdanarson.thorvardur@mayo.edu; 4 Department of Medical Oncology, Royal Free London NHS Foundation Trust and University College London, London WC1E 6BT, UK; nikolaos.diamantis1@nhs.net; 5 Neuroendocrine Tumor Unit, Centre for Gastroenterology, ENETS Centre of Excellence, Royal Free London NHS Foundation Trust and University College London, London WC1E 6BT, UK

**Keywords:** neuroendocrine tumors, somatostatin analogs, combination treatment, carcinoid syndrome

## Abstract

**Simple Summary:**

This comprehensive review explores evolving systemic combination regimens for advanced well-differentiated gastroenteropancreatic neuroendocrine tumors (GEP-NETs) with evidence provided by clinical trials and prospective studies. Phase 1 and 2 trials predominated, with few phase 3 trials impacting clinical practice. Results showed variability in anti-tumor activity of combination regimens, with some showing promising outcomes. Among others, treatments with a peptide receptor radionuclide therapy backbone displayed favorable results and warrant further investigation. In contrast, immune-checkpoint inhibitor-based combinations had limited applicability in this patient population. More extensive trials are needed to determine optimal treatment strategies.

**Abstract:**

There is an evolving landscape of systemic combination regimens for patients with advanced well-differentiated gastroenteropancreatic neuroendocrine tumors (GEP-NETs). In this review, we provide a comprehensive outline of the existing clinical trials/prospective studies investigating these combinations. PubMed was searched using key relevant terms to identify articles referring to GEP-NETs and combination treatments. No systematic search of the literature or metanalysis of the data was performed, and we focused on the most recent literature results. Primarily, phase 1 and 2 clinical trials were available, with a smaller number of phase 3 trials, reporting results from combination treatments across a wide range of antiproliferative agents. We identified significant variability in the anti-tumor activity of the reported combinations, with occasional promising results, but only a very small number of practice-changing phase 3 clinical trials. Overall, the peptide receptor radionuclide therapy (PRRT)-based combinations (with chemotherapy, dual PPRT, and targeted agents) and anti-vascular endothelial growth factor (VEGF) agent combinations with standard chemotherapy were found to have favorable results and may be worth investigating in future, larger-scale trials. In contrast, the immune-checkpoint inhibitor-based combinations were found to have limited applicability in advanced, well-differentiated GEP-NETs.

## 1. Introduction

Neuroendocrine neoplasms (NENs) are considered to be a heterogeneous group of malignancies that can arise at almost any anatomical site, originating from the cells of the diffuse endocrine system [1]. Based on the latest Surveillance, Epidemiology and End Results data, NENs are increasingly recognized as an entity with progressively rising incidence, approximated at 7 cases per 100,000 persons in the United States [2]. NENs most commonly arise in the gastrointestinal tract and are referred to as gastroenteropancreatic (GEP) NENs, with the small bowel, rectum, pancreas, and appendix being the most frequent sites of tumor development [3]. GEP-NENs, like most NENs, also demonstrate significant heterogeneity in terms of tumor differentiation, including well-differentiated GEP neuroendocrine tumors (NETs) and poorly differentiated GEP neuroendocrine carcinomas (NECs), based on the most recent WHO consensus classification [4]. GEP-NETs are further sub-classified by degree of differentiation, mitotic rate (MR—mitoses/2 mm^2^), and Ki-67 proliferation index: G1 (MR < 2, Ki-67 < 3%), G2 (MR 2–20, Ki-67 3–20%), and G3 (MR > 20, Ki-67 > 20%). In addition, poorly differentiated (G3) GEP-NECs (MR > 20 and Ki-67 > 20%) are now considered a distinct category from G3—or high-grade—NETs. [4].

GEP-NETs often tend to be diagnosed at a locally advanced or metastatic stage [5,6,7], with systemic therapies playing a pivotal role in their management. They are frequently characterized by the expression of somatostatin receptors, especially in well- and moderately differentiated tumors (G1 or G2), which has led to the development of several systemic options targeting these receptors [8]. The use of somatostatin analogs (SSAs), such as octreotide and lanreotide, is still the mainstay of treatment for most advanced well-differentiated NETs, with a dual goal of alleviating the symptoms related to the production of vasoactive hormones (e.g., carcinoid syndrome) as well as exerting antiproliferative effects [9,10]. In addition, in the era of precision oncology, multiple targeted agents have emerged as therapeutic options, aiming to disrupt distinct tumorigenesis pathways pertinent to NETs. For example, small molecule tyrosine kinase inhibitors (TKIs), such as sunitinib, are thought to exert their antiproliferative effect via the inhibition of vascular endothelial growth factor (VEGF)-related pathways [11], while the recent addition of peptide receptor radionuclide therapy (PRRT) as a means of targeting radioactive molecules directly to NET cells via expressed somatostatin receptors on their surface has been a major breakthrough [12]. Interestingly, the growing number of antiproliferative agents against NETs via different biologic pathways has paved the way for further clinical investigation and utilization of these drugs as part of several combination regimens, aiming to exert meaningful biologic effects via synergistic mechanisms [13]. The purpose of this review is to provide a comprehensive outline of the current evidence in the field of combination treatments for advanced well/moderately differentiated GEP-NETs, with data derived primarily from pivotal clinical trials and prospective studies in the field.

## 2. Materials and Methods

PubMed was searched using the key terms “neuroendocrine”, “tumors” or “tumours”, “gastroenteropancreatic”, “carcinoid”, “neoplasm”, and “combination” to identify articles referring to GEP-NETs and combination treatments. No systematic search/review of the literature was performed. We had strict inclusion criteria: clinical trials and prospective studies in English, for patients with locally advanced/metastatic, well/moderately differentiated GEP-NETs, and receiving a combination regimen of at least two separate active systemic agents. Of note, we aimed to focus our search on non-cytotoxic chemotherapy combinations, and for that reason, combinations of chemotherapeutic-only agents were excluded. Instead, we included cytotoxic regimens when they were combined with other treatments (targeted, SSAs, PRRT, and immunotherapy). We extracted information, including the combination regimen, tumor differentiation, site of primary tumor, tumor responses per RECIST criteria (partial response—PR, complete response—CR, stable disease—SD, progressive disease—PD, disease control rate or clinical benefit rate—DCR or CBR = PR + CR + SD, and overall response rate—ORR = CR + PR), and survival outcomes, including progression-free survival (PFS) from intervention to PD or death, and overall survival (OS) from intervention to death from any cause. The article references considered to be relevant to the topic were also selected and reviewed, and their data were extracted in the above fashion. No statistical analysis/metanalysis of the data was performed, and we focused on the most recent literature results.

## 3. Results

### 3.1. SSA-Based Regimens (Table 1)

Based on the already-established use of SSA monotherapy in patients with advanced well-differentiated GEP NETs [9,10], there have been multiple studies investigating the combination of SSAs with agents that exert their antiproliferative effect through separate mechanisms of action.

#### 3.1.1. SSAs and IFN-a

Interferon-α (IFN-α) is one of the earliest agents introduced in the treatment of midgut NETs, dating back to 1980s. IFN-α exhibits its antiproliferative effects with induction of expression of several genes implicated in cell cycle arrest (G1-S phase), thus leading to a reduction in tumor growth [14]. The combination of octreotide and interferon has been tested against octreotide monotherapy and has been evaluated in multiple clinical trials, with mixed results. Overall, the addition of IFN-a to the SSA regimen does not seem to provide any additional objective benefit to patients with advanced well-differentiated GEP-NETs and has not been integrated to standard clinical practice.

In a randomized controlled trial by Arnold et al. [15] involving 109 participants with metastatic midgut or pancreatic NETs, patients were randomized to receive either thrice-daily octreotide (study preceded the development of long-acting SSAs) and thrice-weekly IFN-a or octreotide monotherapy. The primary endpoint of the study was median time to treatment failure (PD, intolerable toxicity, patient decision, or death), which was found to be approximately six months for both interventions (*p* = 0.59). The median OS from randomization was 35 months for patients receiving octreotide monotherapy and 51 months for the combination arm (*p* = 0.55). In another randomized prospective study of patients with inoperable/metastatic foregut/midgut and hindgut NETs, 80 study participants were assigned to receive thrice-daily lanreotide, thrice-weekly IFN-a, or a combination of both [16]. The primary endpoint was a 1-year tumor progression rate. After 12 months of treatment, the tumor progression rate was statistically comparable between the groups: 56% in patients treated with either lanreotide or IFN-a and 50% in patients treated with the combination. In another prospective study of 68 patients with midgut carcinoid tumors metastatic to the liver, with prior primary tumor resection [17], participants were randomized to receive octreotide (2–3x daily) or octreotide and IFN-a (3–5 times weekly). The study’s primary endpoints were “time to death” and time to “tumor progression”. The mean 5-year survival rate was 37% in the monotherapy group and 57% in the combination group, but the difference was not statistically significant. Patients treated with octreotide and IFN-α had a significantly lower risk of progressive disease (HR 0.28, 95% CI: 0.16 to 0.45, *p* = 0.008), but the median PFS was not reported.

#### 3.1.2. SSA and Inhibitors of the VEGF Pathway

The VEGF pathway is one of the most commonly implicated pathways in tumorigenesis. The downstream signaling of VEGF is facilitated by a family of receptor tyrosine kinases, leading to upregulation of angiogenesis in the tumor microenvironment, thus promoting tumor growth [18]. The overexpression of VEGF has been implicated in the proliferation of neuroendocrine tumor cells, among other malignancies [19], leading to the increasing utilization of agents targeted against the VEGF pathway. These include monoclonal antibodies binding circulating VEGF, such as bevacizumab, as well multiple different agents known as TKIs, which are active against receptor tyrosine kinases, including VEGFR, with sunitinib being the primary agent used in patients with pancreatic NETs [11]. The antiproliferative effects of the combination of anti-VEGF agents with SSAs has been tested in a series of primarily phase 1 and 2 clinical trials in patients with advanced well-differentiated NETs, as delineated below, and has demonstrated mixed anti-tumor activity overall, with no practice-changing results elicited from these studies.

In a phase 2 study of patients with well-differentiated metastatic carcinoid tumors, who were already receiving octreotide LAR, participants were randomly assigned to receive either bevacizumab or pegylated IFN alfa-2b for 18 weeks, alongside octreotide, followed by a combination of octreotide and bevacizumab and IFN-α after 18 weeks or at disease progression [20]. The primary endpoint of the study was response rate and PFS. Of the 44 patients in the study, 29 patients (66%) had a GEP-NET (24 small bowel, 4 rectum, and 1 gastric), while patients with poorly differentiated carcinoid tumors or NECs were excluded. The DCR was 95% in the bevacizumab arm (no complete responses) and 68% in the IFN arm (only SD). A more favorable PFS rate was observed during the 18 weeks of monotherapy with bevacizumab (95%) versus 68% on the PEG interferon arm (*p* = 0.02). The median PFS by initial treatment assignment was 66 weeks for bevacizumab and 56 weeks (95% CI, 34 to 78) for interferon (*p* = 0.34) for the duration of the study. The lack of statistical significance in the two arms could be expected, given the crossover to the common regimen after 18 weeks of study. Compared with paired baseline measurements on functional CT scans, the authors observed a 49% (*p* = 0.01) and 28% (*p* = 0.01) decrease in tumor blood flow at day 2 and week 18, respectively, among patients treated with bevacizumab. A phase 3 study (SWOG S0518) by the same group [21] re-evaluated the combination of octreotide LAR either with bevacizumab or IFN-a-2b in a larger sample of 427 patients with advanced G1 or G2 midgut or hindgut NETs, with PFS as the primary endpoint. The median PFS assessed by central review was 17 months in the bevacizumab arm and 15.4 months (95% CI, 9.6 to 18.6 months) in the interferon arm (*p* = 0.55). The median OS was 35 months (95% CI, 33.1 to 42.8 months) in the bevacizumab arm and was not reached in the interferon arm (*p* = 0.29). Finally, the combination of octreotide LAR and bevacizumab in conjunction with the HER-2 inhibitor, pertuzumab, was tested for safety and efficacy in a phase 2 prospective study of 43 patients with well/moderately differentiated NETs (mainly GEP-NETs), with the ORR as the primary endpoint [22]. This was a negative study with an ORR of 16% (expected ORR was at least 33%), while median PFS and OS were 6.5 and 26.4 months, respectively.

The combination of octreotide LAR and pazopanib, a multitargeted kinase inhibitor that inhibits VEGF receptors 1, 2, and 3, with octreotide LAR was assessed in a multicenter, single-group, phase 2 study by Phan et al. [23]. Fifty-two patients with G1–G2 carcinoid tumors (mainly small bowel) and pancreatic NETs received a combination of octreotide LAR and daily pazopanib, with an objective response of at least 30% as the primary endpoint of the study. The study did not meet its primary endpoint; however, results of the study demonstrated some clinical activity of the combination for patients with pancreatic NETs but not the NETs of other sites. Seven of thirty-two (22%) patients with pancreatic NETs achieved an objective response; however, no responses were detected in the cohort with carcinoid tumors. The median PFS and OS were 14.4 and 25 months for pancreatic NETs and 12.2 and 18.5 months for the remainder of the carcinoid tumors, respectively.

Another anti-angiogenic TKI, nintedanib, was evaluated in conjunction with octreotide LAR in an open-label, phase 2 trial recruiting patients with locally advanced/metastatic, G1–G2 non-pancreatic NETs, and progressing after a maximum of two lines of therapy, with a 16-week PFS > 40% as the primary endpoint [24]. A total of 32 patients with NETs were enrolled (72% GI), with 30 patients eventually evaluable for the primary endpoint. The primary endpoint was met, with a 16-week PFS rate of 83%, a median PFS of 11 months, and a median OS of 33 months. Only one patient had PR, with 81% of patients achieving SD. However, most patients had some degree of tumor shrinkage (not fulfilling PR criteria) and/or a biochemical response.

#### 3.1.3. SSAs and Inhibitors of Mammalian Target of Rapamycin (mTORi)

MTOR is a serine threonine kinase that stimulates cell growth, proliferation, and angiogenesis, and its inhibition has shown promising results in the field of NETs [25]. Everolimus is a well-established mTORi and currently serves as the standard of care treatment for patients with advanced pancreatic, as well as extra-pancreatic, NETs of the GI and lung (well/moderately differentiated), progressing on prior lines of therapy. The hallmark studies that established everolimus as the standard of care were RADIANT-3 and RADIANT-4, both phase 3 RCTs evaluating the use of everolimus vs. placebo/best supportive care in patients with well/moderately differentiated NETs of the pancreas and GI/lung, respectively [26,27]. In RADIANT-3, everolimus achieved a PFS of 11 months compared with 4.6 months (for the placebo) in patients with pancreatic NETs, with similar results reported by RADIANT-4 (11 months for everolimus vs. 3.9 months for the placebo) for extra-pancreatic NETs. SSAs, in conjunction with everolimus, were used in both studies as the best supportive care in a proportion of the patients (e.g., 40% in RADIANT-3); however, neither study was designed to test the efficacy of the combination but rather the efficacy of everolimus compared with the placebo.

The combination of SSA/mTORi has shown promising clinical benefits overall, although the results from several trials are conflicting. One of the leading trials in the field, RADIANT-2, was a randomized phase 3 study enrolling patients with advanced well/moderately differentiated NETs associated with carcinoid syndrome. In this study, 429 patients were assigned to receive everolimus and octreotide LAR vs. placebo and octreotide LAR [28]. The primary endpoint of the study was PFS between the two arms, with a predefined risk reduction of at least 33% (HR 0.67) in the combination arm. Patients with GEP-NETs (mainly small bowel) comprised the largest bulk of both cohorts. The study demonstrated clinical efficacy of the combination, but it did not achieve its primary endpoint. The median PFS was 16.4 months in the everolimus and octreotide LAR group and 11.3 months in the placebo and octreotide LAR group (one-sided log-rank test; *p* = 0.026), with the combination resulting in a 23% risk reduction (HR 0.77; *p* = 0.026) of progression or death. However, in an ad hoc analysis of RADIANT-2 for colorectal well/moderately differentiated NETs, a significantly longer PFS was achieved with the combination of everolimus and octreotide LAR compared with octreotide LAR monotherapy (30 vs. 7 months, respectively) [29].

In addition, a randomized, open-label, phase 2 study (COOPERATE-2) in patients with advanced, well-differentiated, progressive pancreatic NETs tested the combination of everolimus with a novel SSA agent, pasireotide [30]. Patients were randomized to receive a combination of everolimus and pasireotide LAR or everolimus alone, with PFS as the primary endpoint of the study. No significant difference in PFS was noted between the two arms (16.8 months in the combination arm versus 16.6 months in the everolimus arm), and no significant improvement was observed in the median overall survival (secondary endpoint). The combination of everolimus and octreotide in patients with advanced well/moderately differentiated pancreatic NETs, with or without the addition of the VEGF inhibitor bevacizumab, was evaluated in a randomized phase 2 study (CALGB 80701) by Kulke et al. [31]. Patients were randomized to receive everolimus (*n* = 75) or everolimus and bevacizumab (*n* = 75), while concurrent octreotide LAR was required for all participants. The PFS difference between the two arms was set as the primary endpoint for the study, with a “liberal” target of alpha error < 0.15, with the purpose of screening for the potential efficacy of the combination. The combination of everolimus and bevacizumab elicited a PFS of almost 17 months compared with 14 months for the everolimus monotherapy (*p* = 0.1); the primary endpoint was met, indicating a promising efficacy of the combined regimen that would, nevertheless, require further investigation in a subsequent trial. The ORR was statistically higher in the combination cohort (31% vs. 12%, *p* = 0.005). No difference in OS was noted between the combination and the everolimus monotherapy groups (42.5 vs. 42.1 months, respectively).

**Table 1:** Summary of clinical trials in patients with progressive, advanced GEP-NETs with SSA-based combinations.

Study	Design	Population	Diff	*n*	Regimen	Primary Endpoint	ORR	DCR	PFS and Related Outcomes	**OS and Related Outcomes**
Arnold [15]	Prospective randomized	Pancreatic, midgut	WDNET	109	IFN-α and octreotide vs. octreotide alone	TTF	9% vs. 2%	24% vs. 18%	TTF: median ~6 mos in both arms	Median OS: 51 m vs. 35 m
Faiss [16]	Prospective randomized	Foregut, midgut, hindgut	WDNET	84	Lanreotide or IFN-α or combination	1-year progression rate	4% vs. 4% vs. 7%	32% vs. 30% vs. 25%	1-year progression rate: 56% vs. 56% vs. 50%	-
Kolby [17]	Prospective randomized	Midgut, metastatic to the liver	WDNET	68	IFN-α and octreotide vs. octreotide alone	OS, risk of tumor progression *	-	-	Risk of tumor progression: HR 0.28 (0.16–0.45) * for combination arm	5-year OS: 57% vs. 37%
Yao [20]	Phase 2, two-stage design	Foregut, midgut, hindgut	WDNET	44	Stage 1: Octreotide LAR and BEV or IFN-α-2b. Stage 2: triple therapy	ORR, PFS for Stage 1 (first 18 wks of the study) *	18%% (BEV) vs. 0% (IFN)	95% (BEV) vs. 68% (IFN)	Stage 1 PFS rate 95% vs. 68% * Median PFS (duration of study): 66 vs. 56 wks	1-year, 2-year, and 3-year survival rates; 93%, 67%, and 56%
Yao [21]	Phase 3	Mainly midgut	WDNET	427	Octreotide LAR and either BEV or IFN-α-2b	PFS	13% (BEV) vs. 4% (IFN)	-	Median PFS: 16.6 m (BEV), 15.4 m (IFN)	Median OS: 35.2 m (BEV) vs. NR (IFN)
Bendell [22]	Phase 2	Carcinoids, pancreatic	WDNET	43	Octreotide LAR plus BEV and pertuzumab	ORR (at least 33% required)	16%	53% carcinoids, 23% pancreatic	Median PFS: 12 m (carcinoids), 5.5 m (pancreatic)	Median OS: NR (carcinoids), ~26 m (pancreatic)
Phan [23]	Phase 2	Carcinoid, pancreatic	WDNET	52	Octreotide LAR and pazopanib	ORR (at least 30% required)	22% (pancreatic), 0% (carcinoids)	-	Median PFS: 14.4 (pancreatic), 12.2 (carcinoids)	Median OS: 25 m (pancreatic), 18.5 (carcinoid)
Iyer [24]	Phase 2	Non-pancreatic	WDNET	32	Octreotide LAR and nintedanib	PFS rate > 40% at 16 weeks *	3%	84%	16-week PFS rate: 83% * Median PFS: 11 m	Median OS: 32.7 m
Pavel [28]	Phase 3	Carcinoid, pancreatic	WDNET	429	Octreotide LAR and EVE vs. octreotide LAR	PFS (at least 33% risk reduction) *	2% vs. 2%	86% vs. 83% *	Median PFS: 16.4 m vs. 11.3 m	NR in both arms
Kulke [30]	Phase 2	Pancreatic	WDNET	160	EVE and pasireotide LAR vs. EVE alone	PFS	20% vs. 6%	77% vs. 83%	Median PFS: 16.8 m (combination) vs. 16.6 m	No difference (numbers not provided)
Kulke [31]	Phase 2	Pancreatic	WDNET	150	Octretotide LAR and EVE +/− BEV	PFS (*p* < 0.15) *	31% vs. 12% *	-	Median PFS: 16.7 m vs. 14 m (*p* = 0.1) *	42.1 m vs. 42.5 m

* Indicates statistical significance and/or primary endpoint met. Abbreviations: BEV—bevacizumab, DCR—disease control rate, EVE—everolimus, IFN—interferon, LAR—long acting release, GEP—gastroenteropancreatic, NET—neuroendocrine tumor, NR—not reached, ORR—objective response rate, PFS—progression free survival, OS—overall survival, TTF—time to treatment failure, WDNET—well differentiated neuroendocrine tumor.

### 3.2. PRRT-Based Combinations (Table 2)

#### 3.2.1. PPRT and SSAs

PRRT, as the name implies, is a targeted form of systemic radiotherapy which utilizes the high expression of somatostatin receptors on well-differentiated NETs to facilitate the delivery of radionuclides bound to somatostatin analogues directly to neuroendocrine tumor cells [32]. PRRT in combination with octreotide LAR has now been established as the standard of care in patients with advanced well/moderately differentiated GEP-NETs primarily of midgut origin, based on the results of the NETTER-1 study [12]. NETTER-1 was a phase 3 randomized controlled trial that evaluated the combination of ^177^Lu-Dotatate and octreotide LAR vs. octreotide LAR monotherapy in patients with well-differentiated metastatic midgut NETs progressing on prior lines of therapy, with PFS as the primary endpoint of the study. The combination of ^177^Lu-Dotatate and octreotide LAR led to a significantly superior PFS (not reached at the time of data analysis) compared with 8.4 months in the control group. The ORR (secondary endpoint) was significantly higher (18%) in the ^177^Lu-Dotatate group compared with 3% in the control group (*p* < 0.001). Of note, after the final overall survival analysis was completed in 2021, there was no statistically significant difference in the median OS between the two study arms (48 vs. 36 months, respectively) [33].

#### 3.2.2. Dual PPRT

Based on emerging evidence from the literature, the combination of PRRT agents seems to offer a clinical benefit with similar toxicity to PRRT monotherapy and may warrant further investigation in future trials. In a small phase 2 trial of patients with advanced well/moderately differentiated NETs (mainly GEP-NETs), 26 study participants were assigned to receive 4 therapeutic cycles of alternating ^177^Lu-DOTATATE and ^90^Y-DOTATATE [34]. The primary endpoint was response rate (ORR of at least 40%). The combination induced objective responses in 42.3% of the patients, with a median PFS > 2 years. In addition, there is evidence of superior survival of the PRRT combinations in comparison with monotherapy. In one of the largest prospective cohort studies in the field by Villard [35], patients with metastatic NETs of different sites (including GI carcinoids and pancreatic NETs) were treated with ^90^Y-DOTATOC or with alternating ^90^Y-DOTATOC and ^177^Lu-DOTATOC until tumor progression or permanent toxicity. The main outcomes of interest were the overall survival and severe renal toxicity. By the end of the study, 237 patients completing at least three cycles of ^90^Y-DOTATOC and 249 patients completing at least three cycles of combined treatment were included in the final survival analysis. Patients in the combination cohort had a significantly higher median OS of 5.5 years compared with 4 years in the ^90^Y-DOTATOC monotherapy cohort (*p* = 0.004). The rates of severe renal toxicity (grade 4 and 5) were comparable in both groups (6.9% vs. 6.6%; *p* = 0.93). Similar findings were elicited by Kunikowska et al. in a prospective study of 50 patients with progressive metastatic NETs (foregut (mainly pancreatic), midgut, and hindgut), with SRS-positive disease selected to receive either ^90^Y-DOTATATE or a combination of ^90^Y/^177^Lu-DOTATATE (concurrent single IV administration) [36]. Patients in the combination group had a significantly higher median OS (not reached) compared with patients in the ^90^Y-DOTATATE monotherapy group (26 months, *p* = 0.027). In a similar study by the same authors, 59 patients with progressive advanced GEP-NETs receiving the combination of ^90^Y/^177^Lu-DOTATATE were prospectively followed for 10 years to determine the long-term efficacy and toxicity of the combined regimen [37]. The DCR was noted to approach 90%, with a median PFS of 32 months and an OS of 82 months. In the subgroup analysis, patients with G1 and large bowel NET had the longest PFS/OS. Only one event of myelodysplastic syndrome was noted, occurring 5 years after the PRRT treatment. The rate of nephrotoxicity was 18% (grade 1–3), and no grade 4 nephrotoxicity was observed.

#### 3.2.3. PRRT and mTORi

Clinical data regarding the combination of PRRT treatments with everolimus are scarce. The safety and efficacy of the combination of ^177^Lu-Dotatate and everolimus was assessed in a phase 1 trial of 16 patients with advanced progressive well-differentiated GEP-NETs [38]. The patients received escalating doses of daily everolimus for 24 weeks and PRRT every 2 weeks for 4 cycles. The overall response rate was 44% (7 of 16 patients), and no patient progressed over the 6-month period of treatment. These results indicated the potential benefit of the combination, which needs to be confirmed in future trials.

#### 3.2.4. PRRT and Chemotherapy

The combination of PRRT with chemotherapeutic agents is being studied increasingly more in the field of NETs. In addition to their apparent cytotoxic effects, chemotherapy drugs are thought to exert a synergistic effect with PRRT by acting as radiosensitizers, e.g., by increasing the level of DNA damage or by reducing the efficient DNA replication, thus enhancing the effects of radiation toxicity [39]. So far, no phase 3 trials have been conducted with these combinations; however, evidence from phase 1 and 2 trials show promising ORR/DCR rates as well as clinically meaningful PFS rates, and they may require further investigation in a phase 3 RCT setting.

The efficacy and tolerability of ^177^Lu-Dotatate in conjunction with oral capecitabine was assessed in a phase 2 study of 33 patients with progressive unresectable/metastatic NETs (mainly GEP-NETs) and SRS-positive imaging [40]. The combination appeared to have a clinically meaningful efficacy, with a DCR of 94% (24% PR, 70% SD), while the median PFS and OS had not been reached at a median follow-up of 16 months, and the addition of capecitabine did not seem to significantly increase the overall toxicity of ^177^Lu-Dotatate. A similar combination of ^177^Lu-Dotatate with metronomic capecitabine was evaluated in patients with SRS- and FDG-positive G1–G3 GEP-NETs in a phase 1–2 study by Nicolini et al. [41]. In this study, in which DCR and safety were the primary endpoints, 37 patients were scheduled to receive 5 cycles of PRRT, with metronomic capecitabine between the cycles. The study showed promising results, with a DCR of 85%, while the toxicity profile did not deviate from prior Lu-PRRT trials, with 81% of the patients completing the combination treatment without interruption.

Another phase 1–2 study evaluated the efficacy and safety of the combination of ^177^Lu-Dotatate with oral capecitabine and temozolomide (CAPTEM) in patients with advanced well-differentiated NETs (mainly GEP). Thirty-five patients received PRRT and capecitabine alongside progressively escalating doses of temozolomide. The study had clinically promising results, with a DCR of 91% (15% CR, 38% PR, 38% SD) and a median PFS of 31 months, while the median OS was not reached by the end of the follow-up period [42]. The same combination was evaluated in an open-label parallel group phase 2 trial in patients with advanced midgut or pancreatic NETs [43]. In this trial, 75 patients were randomized (2:1 randomization) to PRRT (^177^Lu-Dotatate) and CAPTEM vs. PRRT (midgut NETs control) or CAPTEM (pancreatic NETs control), with PFS as the primary endpoint. At extended follow-up (~30 months), the study demonstrated superiority in PFS of the combination in patients with pancreatic NETs (compared with CAPTEM alone) but not in patients with midgut NETs (compared with PRRT alone).

A relatively novel PRRT option combining the α-emitting radioisotope actinium-225 with Dotatate (^225^Ac-Dotatate) has been tested in combination with capecitabine as a radiosensitizer in a prospective study of patients with well-differentiated, inoperable, or metastatic GEP-NETs [44]. Here, 91 patients were enrolled (57 with prior ^177^Lu-PRRT), and the median OS was the prespecified primary endpoint. The median OS was not reached, with an OS rate of 71% at 24 months. The median PFS was also not reached, with a 24-month PFS probability of 67.5%.

**Table 2:** Summary of clinical trials in patients with progressive advanced NETs and PRRT-based combinations.

Study	Design	Population	Diff	*n*	Regimen	Primary Endpoint	ORR	DCR	PFS and Related Outcomes	**OS and Related Outcomes**
Strosberg [12]	Randomized phase 3	Midgut	WDNET	229	^177^Lu-Dotatate and octreotide LAR vs. octreotide LAR alone	PFS *	18% vs. 3%	-	NR vs. 8.4 m *	48 m vs. 36 m
Seregni [34]	Phase 2	Carcinoid, pancreatic	WDNET	26	Tandem ^177^Lu-Dotatate and ^90^Y-Dotatate	ORR (at least 40% required), safety	42.3% *	84.6%	25 m	2-year OS: 78%
Villard [35]	Prospective randomized	Carcinoid, pancreatic	WDNET	486	^177^Lu-Dotatoc and ^90^Y-Dotatoc vs. ^90^Y-Dotatoc alone	OS *	21% vs. 16%	45% vs. 31%	-	5.5 y vs. 4 y *
Kunikowska [36]	Prospective	Foregut, midgut, hindgut NETs	WDNET	50	^177^Lu-Dotatate and ^90^Y-Dotatate vs. ^90^Y-Dotatate alone	OS *, EFS	12% vs. 20%	76% vs. 72%	mEFS: 29.4 vs. 21.4 m	NR vs. 26.2 m *
Kunikowska [37]	Prospective	Carcinoid, pancreatic	WDNET	59	^177^Lu-Dotatate and ^90^Y-Dotatate	PFS, OS, DCR	24%	89%	32 m	82 m
Claringbold [38]	Phase 1	GEP	WDNET	16	^177^Lu-Dotatate and EVE	Optimal safety dose	44%	57%	-	2-year OS: 63%
Claringbold [40]	Phase 2	Carcinoid, pancreatic	WDNET	33	^177^Lu-Dotatate and CAP	ORR/DCR, safety	24%	94%	NR (16 m follow-up)	NR (16 m follow-up)
Nicolini [41]	Phase 2	GEP	WDNET	37	^177^Lu-Dotatate and metronomic CAP	DCR	30%	85%	31.4 m (pNETs), 36.1 m (GI-NETs)	
Claringbold [42]	Phase 1/2	Carcinoid, pancreatic	WDNET	35	^177^Lu-Dotatate and CAPTEM	Optimal safety dose	53%	91%	31 m	NR
Pavlakis [43]	Phase 2	Midgut/pancreas	WDNET	75	^177^Lu-Dotatate and CAPTEM vs. PRRT (midgut) or CAPTEM (pNET)	PFS * (for pancreatic only)	-	-	61 vs. 33% at 27 m (pancreatic) 66 vs. 62% at 33 m (midgut)	No difference
Ballal [44]	Prospective	GEP	WDNET	91	^225^Ac-Dotatate and CAP	Median OS	44% (prior PRRT), 44% (naïve)	72% (prior PRRT), 64.6% (naïve)	2-year PFS: 67.5%	2-year OS: 70.8%

* Indicates statistical significance and/or primary endpoint met. Abbreviations: CAP—capecitabine, DCR—disease control rate, EFS-event-free survival, LAR—long acting release, GEP—gastroenteropancreatic, NET—neuroendocrine tumor, NR—not reached, ORR—objective response rate, PFS—progression free survival, OS—overall survival, TEM—temozolomide, WDNET—well differentiated neuroendocrine tumor, PDNEC—poorly differentiated neuroendocrine carcinoma.

### 3.3. Targeted Therapy/Chemotherapy-Based Combinations (Table 3)

#### 3.3.1. Targeted-Therapy Combinations

Based on prior evidence for the single agent efficacy of sorafenib or bevacizumab in patients with advanced GEP-NETs, facilitated via inhibition of the VEGF pathway, an open-label, uncontrolled, multicenter, phase 2 clinical trial evaluated the combination of these two agents in 44 patients with progressive inoperable/metastatic NETs (mainly GEP-NETs) [45]. The primary endpoint of the study was the progression-free survival rate (PFSR) after 6 months of treatment, which was found to be 91%. The DCR was 95%; 10% achieved PR, 85% achieved SD, and two patients (5%) progressed during the treatment period. Despite these results, the combination was associated with unfavorable toxicity, and its use was not recommended.

The safety and preliminary efficacy of the combination of sorafenib with everolimus (mTORi), was assessed in a phase 1 trial by Chan et al. [46], consisting of 21 patients with histologically documented, locally unresectable or metastatic carcinoid, or pancreatic neuroendocrine tumors of low- or intermediate-grade histology. The patients received daily oral everolimus in combination with oral sorafenib twice daily. Among the 17 patients evaluated for radiographic response, the DCR was 82% (6% PR, 76% SD).

The combination of another mTORi, temsirolimus, with bevacizumab was evaluated in a multicenter, single-arm, open-label phase 2 trial of patients diagnosed with advanced well/moderately differentiated pancreatic NETs, progressing on prior lines of therapy [47]. Prior octreotide and/or continued octreotide at a stable dose was allowed, but not any prior therapy with mTOR or VEGF pathway inhibitors. The primary endpoints of the study were the tumor response rate and 6-month PFS. A total of 56 patients were included in the final analysis. The study met its primary endpoint, with a PR rate was 41% (no CR), and a 6-month PFS of 79%. The median OS was 34 months.

#### 3.3.2. Chemotherapy and Anti-VEGF Combinations

The utilization of anti-VEGF agents (e.g., bevacizumab) with the propensity to induce tumor hypoxia alongside cytotoxic agents has been tested extensively in a wide range of tumors and is now the standard of care in other GI malignancies, such as metastatic colorectal adenocarcinoma [48]. There have been several studies extrapolating from this paradigm, primarily phase 2 studies in the field of advanced NETs, which looked into the efficacy of cytotoxic chemotherapy when combined with regimens that inhibit the VEGF pathway. Overall, the combinations of agents, such as fluoropyrimidines, oxaliplatin, and bevacizumab, appear to have some benefit in patients with advanced well-differentiated NETs progressing on prior lines of treatment, thus warranting further assessment in larger trials.

**Table 3:** Summary of clinical trials in patients with locally advanced/metastatic GEP-NENs with targeted therapy and chemotherapy combinations.

Study	Design	Population	Grade	*n*	Regimen	Primary Endpoint	ORR	DCR	PFS and Related Outcomes	**OS and Related Outcomes**
Castellano [45]	Phase 2	Carcinoid, pancreatic	WDNET	44	Sorafenib and bevacizumab	Efficacy (PFS rate), safety	10%	95%	PFS rate 91%, median PFS: 12.4 m	-
Chan [46]	Phase 1	Carcinoid, pancreatic	WDNET	21	Sorafenib and everolimus	Safety, preliminary efficacy (ORR)	6%	82%	-	-
Hobday [47]	Phase 2	Pancreatic	WDNET	56	Temsirolimus and bevacizumab	Efficacy (ORR, PFS), safety	41% *	-	6-month PFS: 79%, median PFS 13.2 m *	Median OS: 34 m
Mitry [49]	Phase 2	Non-pancreatic	WDNET	49	CAP and BEV	PFS *	18%	88%	Median PFS: 23.4 m*	2-year OS: 85%
Ducreux [50]	Phase 2	Pancreatic	WDNET	34	5FU/STZ and BEV	PFS *	56%	100%	Median PFS: 23.7 m *	2-year OS: 88%
Chan [51]	Phase 2	Carcinoid, pancreatic	WDNET	34	TEM and BEV	Efficacy (ORR), toxicity	15% (all pancreatic)	80%	Median PFS: 11 m	Median OS: 33 m
Bhave [52]	Phase 1/2	Pancreatic	WDNET	28	TEM and pazopanib	Efficacy (ORR), toxicity	25%	70%	Median PFS: 12 m	Median OS: 36 m
Kunz [53]	Phase 2	Carcinoid, pancreatic	Mainly WDNETs, PDNECs	36	mFOLFOX and BEV	ORR, PFS, toxicity	31%	95%	Median PFS: 21 m	-
Kunz [53]	Phase 2	Carcinoid, pancreatic	WDNETs, PDNECs	40	CAPOX and BEV	ORR, PFS, toxicity	17%	77%	Median PFS: 16.7 m	-
Berruti [54]	Phase 2	Carcinoid, pancreatic	WDNET	45	Metronomic CAP and BEV and octreotide LAR	ORR	18%	82%	Median PFS: 15 m	Median OS: NR
Koumarianou [55]	Phase 1	Carcinoid, pancreatic	WDNET	15	Metronomic TEM and BEV and octreotide LAR	Efficacy (ORR, TTP), safety	64%	85%	Median TTP: 36 weeks	-
Grande [56]	Phase 2	Pancreatic	WDNET	17	Sunitinib and evophosphamide	ORR	17.6%	82%	10.4 m	NR

* Indicates statistical significance and/or primary endpoint met. Abbreviations: BEV—bevacizumab, CAP—capecitabine, DCR—disease control rate, LAR—long acting release, GEP—gastroenteropancreatic, NR—not reached, ORR—objective response rate, OX—oxaliplatin, PFS—progression free survival, OS—overall survival, TTP—time to progression, TEM—temozolomide, WDNET—well differentiated neuroendocrine tumor, PDNEC—poorly differentiated neuroendocrine carcinoma.

In a phase 2 non-randomized study (BETTER trial), the efficacy and tolerability of capecitabine and bevacizumab was tested in a cohort of 49 patients with progressive, metastatic, well-differentiated GI-NETs (non-pancreatic) (Ki-67 < 15%) and no prior systemic chemotherapy [49]. The primary endpoint of the study was PFS. The study met its primary endpoint, with a median PFS of 23.4 months. The CR was 88% (18% PR, 70% SD). The median OS was not reached, and the 2-year survival rate was 85%. The most frequent grade 3–4 adverse events were hypertension (31%), diarrhea (14%), and hand–foot syndrome (10%). The authors also conducted a separate phase 2 trial that included 34 patients with metastatic well-differentiated pancreatic NETs with the same name (BETTER), this time utilizing the combination of 5FU/streptozocin with bevacizumab, with PFS as the primary endpoint of the study [50]. The primary endpoint was met; after a median follow-up of 24 months, the median PFS was 23.7 months, with a 100% DCR (56% PR, 44% SD). The 2-year OS rate was 88%. The most frequent grade 3–4 adverse events were hypertension (21%), abdominal pain (12%), and thromboembolic events (9%). The combination of temozolomide and bevacizumab was assessed in a phase 2 trial recruiting patients diagnosed with carcinoid tumors or pancreatic NETs [51]. Thirty-four patients (56% with carcinoid and 44% with pancreatic NETs) were included in the study. The primary endpoint was the radiographic response rate defined by RECIST. The DCR was 80% (15% PR, 65% SD). The patients with pancreatic NETs appeared to have a more favorable response, with a PR of 33%, compared with those with carcinoid tumors (0%). The median PFS was 11.0 months (14.3 months for pancreatic NETs vs. 7.3 months for carcinoid tumors). The efficacy (ORR) and tolerability of pazopanib and temozolomide was assessed in an open-label phase 1/2 study of patients with progressive, advanced, well-differentiated pancreatic NETs [52]. A total of 28 patients (20 evaluable) were included in the study. The best ORR was PR in five patients (25%) and SD in nine patients (45%), and the DCR was 70%.

The combination of FOLFOX or CAPEOX with bevacizumab in patients with carcinoid tumors and pancreatic NETs was tested in two separate phase 2 trials (FOLFOX/B, CAPOX/B) by Kunz et al. [53]. Both studies included advanced NENs progressive on prior lines of treatment, including chemotherapy, regardless of grade or site. Patients were stratified according to tumor type into a carcinoid cohort, a pancreatic NET cohort, or a poorly differentiated NEC cohort. Most study participants had well/moderately differentiated tumors, two patients had NEC in the FOLFOX/B study as did four in the CAPOX/B study. The concurrent use of octreotide was allowed in up to 43–44% of the patients. The primary endpoint was ORR for both studies. The study was designed in a Simon’s two-stage fashion; however, stage 2 was never completed due to poor accrual, and neither study was considered to have met the primary endpoint. In the FOLFOX/B study, 36 patients received modified FOLFOX and bevacizumab. The ORR was 31% (all PR), and the DCR was 95% (31% PR, 64% SD), with a median PFS of 21 months. In the CAPOX/B study, 40 patients were assigned to receive capecitabine and oxaliplatin alongside bevacizumab. The ORR was 17% (only PR), the DCR was 77% (17% PR, 60% SD), and the median PFS was 16.7 months. Despite its being a negative study, these combinations appeared to confer clinically meaningful tumor responses and stabilization of disease, suggesting that selected patients with NET may benefit from these regimens.

Cytotoxic chemotherapy has also been tested in conjunction with anti-VEGF agents and SSAs. More specifically, in a prospective phase 2 study that included 45 patients with well/moderately differentiated NETs arising from various primary sites, the study participants were assigned to receive monthly octreotide LAR, bevacizumab every 2 weeks, and capecitabine in a metronomic daily dose [54]. The primary endpoint of the study was tumor response rate. According to the intent-to-treat analysis, a partial response was observed in 8/45 patients (18%), while patients with pancreatic NETs had a higher response rate (26%) compared with those in non-pancreatic sites (11.5%), while the median PFS was 15 months in the entire cohort. In a similar prospective study by Koumarianou et al. [55], 15 patients with well/moderately differentiated NETs of primarily GEP origin received daily oral temozolomide alongside bevacizumab (every 3 weeks) and monthly octreotide LAR, with the toxicity, efficacy (radiologic tumor response), and time-to-progression as the primary endpoints of the study. The DCR was noted to be 85%, with most patients achieving a PR (57%). The median time to progression was 36 weeks. Another interesting trial (SUNEVO) also investigated the hypothesis that the hypoxia induced by the multi-TKI sunitinib via inactivation of the VEGF pathway might facilitate the activation of the prodrug evofosfamide in patients with WDNETs [56]. This phase 2 study recruited 17 systemic treatment-naïve patients with advanced well-differentiated pNETs with a Ki67 ≤ 20%, who were assigned to receive the above combination. The ORR was the primary endpoint for the study. At a median follow-up of 15.7 months, the ORR was 17.6% (one CR and two PR), and 64.7% exhibited SD. The median PFS was determined to be 10.4 months. The combination was found to have a considerable toxicity profile, with 64.7% of patients experiencing grade 3 or higher treatment-associated adverse events.

### 3.4. Immune-Checkpoint Inhibitors (ICIs) and Combinations (Table 4)

#### 3.4.1. Dual ICIs

ICIs are monoclonal antibodies directed against molecules such as programmed cell death protein-1 and its ligand (PD-(L)1), cytotoxic T-lymphocyte–associated antigen 4 (CTLA-4) which control immune surveillance. They areconsidered to be a revolutionary treatment option in the field of primarily solid tumors and are increasingly utilized in multiple tumor types. However, to date, they have shown a limited benefit in the field of low grade well-differentiated NETs, with their use reserved primarily for higher grade G3 NETs and poorly-differentiated NECs.

**Table 4:** Summary of clinical trials in patients with locally advanced/metastatic GEP-NENs with ICI-based combinations. (All comparisons are not statistically significant unless otherwise indicated).

Study	Design	Population	Grade	*n*	Regimen	Primary Endpoint	ORR	DCR	PFS and Related Outcomes	**OS and Related Outcomes**
Klein [57]	Phase 2	NENs of all sites (mainly lung/GEP)	WDNET/PDNEC	29	Ipilimumab/Nivolumab induction→Nivolumab maintenance	CBR (CR + PR + SD)~DCR	24%	74%	Median PFS: 4.8 m	Median OS: 14.8 m
Patel [58]	Phase 2	Non-pancreatic NENs	WDNET/PDNEC	32	Ipilimumab/Nivolumab	ORR	25% (no responses in WDNETs)	65%	6-month PFS: 31%	Median OS: 11 m
Capdevilla [59]	Phase 2	C1: lung carcinoids C2: G1–2 GI carcinoids C3: G1–2 pancreatic C4: G3 GEP	G1–3 NENs	123	Durvalumab/Tremelimumab	CBR (CR + PR + SD) for G1/2 NETs, 9-month OS for G3 NENs *	C1: 7.4% C2: 0% C3: 6.3% C4: 9.1%	C1: 7.4% C2:32.3% C3: 25%	-	9-month OS: 36% *
Owen [60]	Phase 2	GI/lung NENs	WDNET/PDNEC	28	Nivolumab and TEM	ORR	32%	-	Median PFS: 8.8 m	Median OS: 32.3 m
Halperin [61]	Single-arm, open-label nonrandomized	Carcinoids, pancreatic	WDNET	40	Atezolizumab and BEV	ORR	18% overall, 20% (pancreatic), 15% (non-pancreatic)	-	Median PFS: 14.9 m (pancreatic), 14.2 m (non-pancreatic)	-

* Indicates statistical significance and/or primary endpoint met. Abbreviations: BEV—bevacizumab, CBR—clinical benefit rate, DCR—disease control rate, GEP—gastroenteropancreatic, NET—neuroendocrine tumor, ORR—objective response rate, PFS—progression free survival, OS—overall survival, TEM—temozolomide, WDNET—well differentiated neuroendocrine tumor, PDNEC—poorly differentiated neuroendocrine carcinoma.

Immunotherapy using combined anti-PD-1 and anti-CTLA-4 in the field of NETs has been investigated in two different studies, CA209-538 and DART SWOG 1609, both enrolling patients with metastatic rare cancers. CA209-538 was a multicenter, open-label, phase 2 study evaluating the combination of nivolumab (anti-PD-1) and ipilimumab (anti-CTLA-4) in patients with metastatic rare malignancies [57]. The study comprised an induction phase with combined nivolumab/ipilimumab every three weeks for four doses, followed by maintenance nivolumab every 2 weeks until progression or unacceptable toxicity. The primary endpoint was the clinical benefit rate (PR, CR, and SD) according to the RECIST criteria. Among others, twenty-nine patients with NENs were finally included, ranging from low to high grade NETs and high-grade NECs. Most patients had an NEN of lung origin (39%), 36% had GEP-NENs, and the remainder had NENs of other sites. Of note, patients with small cell histology were excluded. The ORR of the entire cohort was 24%, and SD was noted in 48%, leading to a DCR of 72%. Of note, the highest benefit was observed in the high grade NENs (NETs and NECs), reaching an ORR of 31%, with the intermediate grade NENs reaching an ORR of 23%. No responses were noted in patients with low-grade tumors. The median PFS and OS were 4.8 months 14.8 month, respectively.

The same combination was also evaluated as part of the DART SWOG 1609 study, a prospective, open-label, multicenter phase 2 clinical trial across multiple rare tumor cohorts. A subgroup analysis of that study provided data on outcomes for the cohort of non-pancreatic NENs [58]. The primary endpoint was ORR. Thirty-two patients with NENs were included in the study (56% high-grade disease). The most common primary sites were the GI (47%) and the lung (19%). The ORR was 25% in the entire cohort. In subgroup analyses, the patients with high-grade NECs had an ORR of 44% compared with 0% in low/intermediate-grade tumors (*p* = 0.004). The 6-month PFS was 31%, and the median OS was 11 months. The results of these studies led to a level 2B recommendation by NCCN for the use of ipilimumab/nivolumab as a reasonable treatment option for patients with locally advanced and metastatic G3 NETs of unfavorable biology (Ki-67 > 55, negative SSA imaging) and poorly differentiated NECs progressing on prior lines of therapy. The interpretation of the results of CA209-538 and DART is challenging, as there was no separation of well-differentiated and poorly differentiated neuroendocrine neoplasms.

Another combination of anti-PD-L1/CTLA-4 agents, durvalumab and tremelimumab, was assessed in a multi-cohort phase 2 study of patients with advanced NENs of gastroenteropancreatic or lung origin (DUNE trial) [59]. Patients were recruited in four cohorts—typical/atypical lung carcinoids (C1), G1/2 gastrointestinal (C2), G1/2 pancreatic (C3), and G3 NENs of gastroenteropancreatic origin (C4)—after progression on prior lines of treatment. Patients received durvalumab and tremelimumab every 4 weeks. The primary endpoint was a 9-month CBR for C1–3 and a 9-month OS rate for C4. The study had mixed results, not meeting the primary endpoint for C1–3, with CBRs of 7.4%, 32.3%, and 25%, respectively, while meeting the primary endpoint for C4, with a 9-month OS rate of 36%. Given the OS results, this regimen may require further assessment in future trials, especially for the high-grade populations.

#### 3.4.2. Miscellaneous ICI-Based Combinations

The combination of nivolumab with the alkylating agent temozolomide was assessed in a phase 2 trial recruiting patients with advanced NENs (G1-G3 NETs as well as NECs). Fifty percent were of GEP origin [60]. The ORR was set as the primary endpoint of the study, with 35% or more considered as promising. Among all 28 patients evaluated (50% GEP origin, 18% high-grade), the ORR was 9/28 (32%). The ORR was higher in the lung NENs (64%) compared with other sites. The median PFS was 8.8 months, and the median OS was 32.3 months.

The combination of anti-PD-L1 agent atezolizumab with bevacizumab has been evaluated in a single-arm, open-label, nonrandomized clinical study in patients with rare cancers, which included 40 patients with advanced, progressive G1/G2 NETs (50% pancreatic, 50% extra-pancreatic NETs), with the ORR as the primary endpoint of the study [61]. The ORR was observed in a total of 7/40 patients (18%): 4/20 with pancreatic NETs (20%) and 3/20 (15%) with non-pancreatic NETs. The median PFSs were 14.9 months and 14.2 months, respectively, in these cohorts.

### 3.5. Studies in Progress

Combination regimens for patients with advanced well-differentiated GEP-NETs are considered a promising way of moving forward, with several clinical trials in-progress investigating a variety of agents. Notable mentions include the combination of newer TKIs, such as cabozantinib or lenvatinib, alongside SSAs, chemotherapy, PRRT, ICIs and everolimus; combinations of ^177^Lu-PRRT with chemotherapy, ICIs as well as targeted agents with mechanisms of action that have not been yet investigated in NETs, such as PARP inhibitors (olaparib), DNA-dependent protein kinase inhibitors (peposertib), and triapine, a ribonucleotide reductase inhibitor. A summary of the ongoing trials can be found in Supplementary Table S1.

## 4. Conclusions and Future Directions

Our comprehensive review revealed a multitude of clinical trials—mostly phase 1 and 2 trials, with fewer phase 3 trials—that explored the outcomes of combining various antiproliferative agents to treat advanced well-differentiated GEP-NETs. The results of these combinations varied considerably in terms of their efficacy against tumors, with occasional promising outcomes, but only a few phase 3 clinical trials have so far been conducted with a potential to impact clinical practice. Overall, apart from the already-established ^177^Lu-Dotatate/octreotide LAR combination as the standard of care based on the results of the NETTER-1 trial, the PRRT-based combinations (with chemotherapy, dual PPRT, and targeted agents) appear to have a promising potential to yield meaningful results in the future and are (not surprisingly) the subject of investigation in several ongoing clinical trials. In addition, anti-VEGF agents, such as bevacizumab, appear to have some clinical benefit when combined with standard chemotherapy, while the addition of anti-VEGF agents to SSAs appears less likely to confer additional anti-tumor activity. The same likely holds true for the combinations of SSA/IFN-a and SSA/TKI regimens. Similarly, ICI-based combinations were found to have limited applicability in advanced well-differentiated GEP-NETs, although they appear to have a role in the management of higher-grade NETs and NECs. The combinations of ICIs with PRRT would also be an interesting consideration, and there is a clinical trial currently investigating that (Supplementary Table S1). In conclusion, the role of combination treatments in patients with WD GEP-NETs is relatively promising, and the results of trials in-progress are eagerly awaited to elucidate further the clinical utility of this approach, given that the increasing incidence and improved international collaborations now permit the design and conduct of such trials, first and foremost in the interest of our patients [62].

## Data Availability

Not applicable.

## Supplementary Materials

https://www.mdpi.com/article/10.3390/biology12081069/s1, Table S1: Trials in development with combination regimens in patients with well/moderately differentiated GEP-NETs.
