# Supplementary material for: Combination Systemic Therapies in Advanced Well-Differentiated Gastroenteropancreatic Neuroendocrine Tumors (GEP-NETs): A Comprehensive Review of Clinical Trials and Prospective Studies
Source: Biology (Basel). 2023 Jul 30;12(8):1069. doi: 10.3390/biology12081069 (PMC10452098; doi:10.3390/biology12081069)
Supplement: Supplementary file 1 [file biology-12-01069-s001.zip › biology-2404994-supplementary.pdf]

Supplementary Table S1. Trials in development with combination regimens in patients with well/moderately differentiated GEP-NETs.

Study	Design	Population	Grade	Regimen	Primary Endpoint
**NCT05048901**	Phase 1/2	GEP-NETs	G1-G2	Cabozantinib plus lanreotide	MTD, PFS
**NCT04893785**	Phase 2	GEP-NETs	G1-G3	Cabozantinib plus temozolomide	ORR
**NCT04400474**	Phase 2 basket trial	WDNETs, NECs, anaplastic thyroid, pheo/para, adrenocortical carcinoma	G1-G3	Cabozantinib plus atezolizumab	ORR
**NCT03950609**	Phase 2	Carcinoid tumors	G1-G2	Lenvatinib plus everolimus	ORR
**NCT03290079**	Phase 2	WDNETs	G1-G2	Lenvatinib plus pembrolizumab	ORR
**NCT03070301**	Phase 2	Foregut	G1-G2	Ribociclib plus everolimus	PFS
**NCT02358356**	Two parallel Phase 2 trials	Trial 1: Pancreatic<br>Trial 2: Midgut SRS+	G1-G2	CAPTEM plus ^{177}Lu-Dotatate vs. CAPTEM (pancreatic) or ^{177}Lu-Dotatate (midgut)	PFS
**NCT02736448**	Randomized phase 2 parallel group study	GEP-NETs, SRS+	G1-G3	Arm 1: ^{177}Lu-Dotatate PLUS CAP followed by SSA-LAR<br>Arm2: ^{177}Lu-Dotatate followed by SSA-LAR	PFS
**NCT04750954**	Phase 1b	Pancreatic, SRS+	G1-G2	Peposertib plus ^{177}Lu-Dotatate	Safety and MTD
**NCT04234568**	Phase 1	GEP-NETs, SRS+	G1-G2	Triapine plus ^{177}Lu-Dotatate	Safety and MTD
**NCT04086485**	Phase 1/2	GEP-NETs, SRS+	G1-G2	Olaparib plus ^{177}Lu-Dotatate	Safety and MTD
**NCT05249114**	Phase 1	GEP-NETs, SRS+	G1-G2	Cabozantinib plus ^{177}Lu-Dotatate	Safety and MTD
**NCT04614766**	Phase 1/2	Midgut NETs, pheo, para	G1-G2	^{131}I-Iobenguane plus ^{177}Lu-Dotatate	MTD, ORR
**NCT04525638**	Phase 1	Pancreatic	G1-G2	Sunitinib plus ^{177}Lu-Dotatate	Safety
**NCT04525638**	Phase 2	G3 WDNETs, PDNECs	G3	^{177}Lu-DOTATATE plus nivolumab	ORR

Abbreviations: BEV – bevacizumab, CAP – capecitabine, CBR – clinical benefit rate, DCR – disease control rate, GEP – gastroenteropancreatic, MTD – maximal tolerated dose, NET – neuroendocrine tumor, NR – not reached, ORR – objective response rate, OS – overall survival, PFS – progression free survival, SSA - somatostatin analogue, SRS – somatostatin receptor scintigraphy, TEM – temozolomide, WDNET – well differentiated neuroendocrine tumor, PDNEC – poorly differentiated neuroendocrine carcinoma.
